# IGFBP2 from a novel copper metabolism-associated biomarker promoted glioma progression and response to immunotherapy

**DOI:** 10.3389/fimmu.2023.1282734

**Published:** 2023-10-19

**Authors:** Qisheng Luo, Junhong Zhuang, Dandan Zheng, Changfeng Miao, Hongcheng Luo, Jun Peng, Chuanhua Zheng, Chengjian Qin, Chuanliu Lan, Meiqin Chen, Ying Xia, Deyou Huang, Zigui Chen

**Affiliations:** ^1^Department of Neurosurgery, Affiliated Hospital of Youjiang Medical University for Nationalities, Baise, Guangxi, China; ^2^Department of Neurology, Affiliated Haikou Hospital of Xiangya Medical School, Central South University, Haikou, China; ^3^Department of Radiation Oncology, The First Affiliated Hospital Zhejiang University, Hangzhou, China; ^4^Department of Laboratory Medicine, Neurosurgery Second Branche, Hunan Provincial People’s Hospital (The First affiliated Hospital of Hunan Normal University), Changsha, Hunan, China; ^5^Department of Laboratory Medicine, Affiliated Hospital of Youjiang Medical University for Nationalities, Baise, Guangxi, China; ^6^Department of Neurosurgery, Affiliated Haikou Hospital of Xiangya Medical School, Central South University, Haikou, China; ^7^Department of Radiation Oncology, Affiliated Jinhua Hospital, Zhejiang University School of Medicine, Jinhua, Zhejiang, China; ^8^Department of Radiology, Affiliated Hospital of Youjiang Medical University for Nationalities, Baise, Guangxi, China

**Keywords:** gliomas, copper metabolism, nucleotide metabolism, immunotherapy, IGFBP2

## Abstract

**Introduction:**

Copper metabolism encompasses all cellular metabolic processes involving copper ions and plays a significant role in the pathogenesis of diseases, including cancer. Furthermore, copper is intricately involved in various processes related to nucleotide metabolism. However, a comprehensive analysis of copper metabolism in gliomas remains lacking despite its importance.

**Methods:**

To address this gap, glioma patients were stratified based on the expression levels of copper metabolism-related genes. By utilizing machine learning techniques, a novel copper metabolism-associated biomarker was developed. The potential of this biomarker in prognosis, mutation analysis, and predicting immunotherapy response efficiency in gliomas was systematically investigated.

**Results:**

Notably, IGFBP2, identified as a glioma tumor promoter, was found to promote disease progression and influence immunotherapy response. Additionally, glioma-derived IGFBP2 was observed to enhance microglial migration. High IGFBP2 expression in GBM cells facilitated macrophage interactions through the EGFR, CD63, ITGB1, and CD44 signaling pathways. Discussion: Overall, the copper metabolism-associated biomarker shows promising potential to enhance the clinical management of gliomas, offering valuable insights into disease prognosis and treatment strategies.

## Introduction

Copper metabolism encompasses all cellular metabolic processes involving copper ions, including pathways and proteins that require copper ions to function as active enzymes (1). Genetic diseases related to copper metabolism deficiency, such as Wilson disease and Menkes disease, have been extensively studied and are known to be associated with symptoms such as growth retardation, hypopigmentation, neurodegenerative diseases, and connective tissue defects (2). The liver, an organ with active copper metabolism, plays a pivotal role in transporting excess copper ions from the small intestine to the liver through the blood. These copper ions are then stored within liver cells. It is important to note that impairment of copper metabolism often manifests first in liver dysfunction (3). Additionally, copper metabolism has been closely associated with cancer, particularly concerning mutations in copper ion transporters (4).

The relationship between copper metabolism and nucleotide metabolism is rooted in the dependency of certain enzymes involved in nucleotide synthesis on copper ions. Ribonucleotide reductase, a critical enzyme affected by copper, converts ribonucleotides (RNA building blocks) into deoxyribonucleotides (DNA building blocks), essential for DNA synthesis and repair. Copper serves as an important electron carrier for ribonucleotide reductase, facilitating the conversion of ribonucleotides to deoxyribonucleotides by generating superoxide radicals through copper-dependent enzymes like copper-zinc superoxide dismutase (CuZnSOD). This process is crucial for maintaining proper nucleotide levels and ensuring accurate DNA replication and repair (4).

In the context of gliomas, copper metabolism has emerged as an important player. STEAP2, a copper-associated protein, has been linked to glioma prognosis and immune infiltration (5). Iron and copper complexes have been reported to possess antioxidant activity, inhibiting the metastatic potential of glioma cells (6). The copper coordination compound Cas III-La exhibits anti-proliferative, pro-apoptotic, and anti-invasive effects in glioma cells, mediated through reactive oxygen species and the Wnt/β-catenin pathway (7). Additionally, enhanced Copper-Temozolomide interactions have shown promise in chemotherapy against glioblastoma (8). Despite these findings, a comprehensive analysis of copper metabolism in gliomas remains lacking.

To address this gap, machine learning was employed in this study to group glioma patients based on the expression levels of copper metabolism-related genes. This approach facilitated the development of a copper metabolism-associated biomarker. The study systematically explored the prognostic value, mutation features, and immunotherapy response efficiency associated with this biomarker (9, 10). Notably, IGFBP2 was identified as a tumor promoter in gliomas, influencing tumor cell proliferation and migration, and also contributing to microglia migration.

In summary, copper metabolism plays a significant role in various cellular processes and has implications for disease pathogenesis, including cancer. In gliomas, copper metabolism has been associated with disease progression and potential therapeutic opportunities. A copper metabolism-associated biomarker was developed using machine learning, providing insights into its prognostic value and therapeutic relevance in gliomas. Further research in this area can potentially improve clinical management and treatment strategies for glioma patients.

## Methods

### Data collection

Glioma patients’ transcriptome data and clinical information were accessed from two publicly available databases: The Cancer Genome Atlas (TCGA) and Chinese Glioma Genome Atlas (CGGA). These datasets were subsequently used in the follow-up bioinformatics analysis.

### Identification of copper metabolism-related clusters

The kmdist approach from the R package ConsensusClusterPlus was used to determine the copper metabolism-related clusters based on the copper metabolism-related genes. Principal component analysis (PCA) was then used to demonstrate the efficiency of these clusters. Survival curves between the two copper metabolism-related clusters were also generated using the R package survival.

### Construction of the copper metabolism-associated biomarker

Differential expression analysis was performed to identify the differentially expressed genes (DEGs) between the two clusters related to copper metabolism. Prognostic DEGs were determined using univariate Cox regression analysis. To reduce dimensions and create the copper metabolism-associated biomarker, the Random Survival Forest algorithm was utilized, followed by the least absolute shrinkage and selection operator (LASSO) algorithm. The expression value of each gene was multiplied by its corresponding coefficient to calculate the copper metabolism-associated biomarker. Subsequently, survival curves based on the groups defined by the copper metabolism-associated biomarker were generated using the R package survival. The receiver operating characteristic (ROC) curves related to the biomarker were plotted using the R package pROC. Univariate and multivariate Cox regression analyses were conducted to evaluate the prognostic value of the copper metabolism-associated biomarker in ten glioma cohorts.

### Mutation analysis of the copper metabolism-associated biomarker

To identify significantly amplified or deleted copy number variations (CNVs), GISTIC 2.0 was employed. GISTIC 2.0 is a computational tool that analyzes DNA copy number data to identify recurrently altered regions in the genome. It uses statistical methods to determine the significance of the alterations and assigns a score to each region based on its frequency and amplitude of alteration.

In addition to CNVs, altered single nucleotide polymorphisms (SNPs) were identified using the R package maftools. SNPs are variations in a single nucleotide base in the DNA sequence. The maftools package is a bioinformatics tool that allows for the analysis and visualization of mutation data from cancer genomic studies. It can be used to identify and characterize SNPs that are associated with cancer development or progression.

### Immunotherapy response efficiency analysis and functional annotation of the copper metabolism-associated biomarker

The correlation between the copper metabolism-associated biomarker and immune infiltrating cells was analyzed using the MCPcounter algorithm (11), ssGSEA algorithm (12), and TIMER algorithm (13). Moreover, immune modulators were also investigated. T cell-inflamed gene expression profile (GEP), Cytotoxic activity (CYT), and Tumor mutation burden (TMB) data were collected from previous studies (14–16). Furthermore, the Tumor Immune Dysfunction and Exclusion (TIDE) analysis was utilized to estimate immunotherapy responses (17).

### Drug prediction of the copper metabolism-associated biomarker

Candidate chemotherapy agents were predicted using the R package “oncoPredict” (18).

Immunotherapy response efficiency analysis and functional annotation of IGFBP2.

The R package survival was utilized to generate survival curves for two gene-based groups, which consisted of ABCC3, AL161787.1, EMP3, and IGFBP2. Additionally, the study examined the correlation between IGFBP2 and immune modulators. Furthermore, gene set enrichment analysis (GSEA) was performed on the copper metabolism-associated biomarker using gene ontology (GO) and Kyoto Encyclopedia of Genes and Genomes (KEGG) terms.

### Single-cell RNA sequencing (sc-RNAseq) analysis

The SCP50 and SCP393 datasets were obtained from the Single Cell Portal platform. Cells with the most prominent gene alterations were integrated using the “FindClusters” algorithm. The annotation of malignant cells was performed using the R package “CopyKAT” (19). Using the R package “scCATCH”, non-cancerous cell clusters were defined (20). The sc-RNA seq data were processed using the R package “Seurat” (21), and cell communication clusters were determined using the R package “iTalk”.

The detailed methods for *in vitro* validation are provided in the **Supplementary Materials**.

## Results

### The copper metabolism-related clusters could discriminate survival outcomes

**Figure 1:**
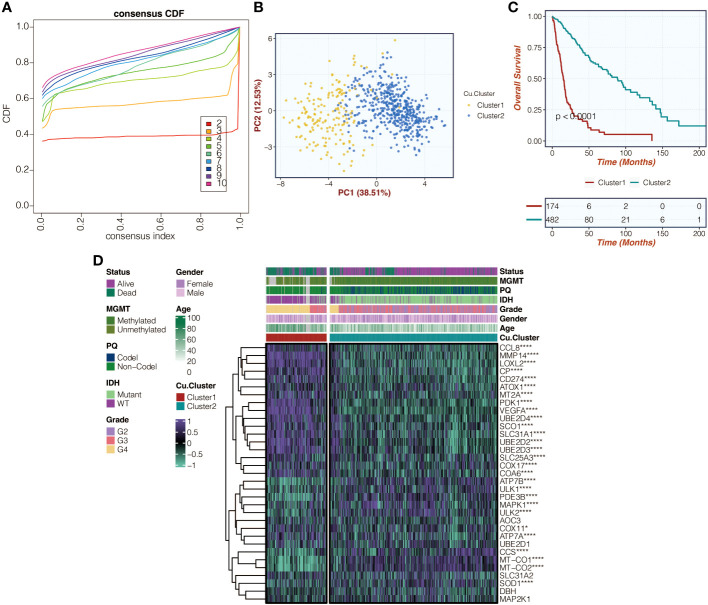
Illustration of copper metabolism-related clusters. **(A)** Presentation of consensus CDF curves depicting copper metabolism-related clusters within the TCGA cohort. **(B)** PCA was used to analyze copper metabolism-related clusters in the TCGA cohort. **(C)** Survival curves related to copper metabolism clusters in the TCGA cohort were examined. **(D)** A heatmap was created to display expression variations of copper metabolism-related genes within the identified copper metabolism-related clusters in the TCGA cohort. *, P<0.05; ****, P<0.0001.

**Figure 1A** in the study shows the consensus cumulative distribution function (CDF) curves of the copper metabolism-related clusters in the TCGA cohort. The curve appears to be remarkably smooth with a k value of 2. **Figure 1B** displays these clusters’ principal component analysis (PCA) results, showing distinct separation of glioma patients based on the copper metabolism-related clusters. **Figure 1C** presents the survival curves, indicating significant differences in survival outcomes among glioma patients within these clusters. Lastly, **Figure 1D** illustrates the even distribution of expression differences of copper metabolism-related genes within the copper metabolism-related clusters.

### The copper metabolism-associated biomarker has a significant prognostic value

**Figure 2:**
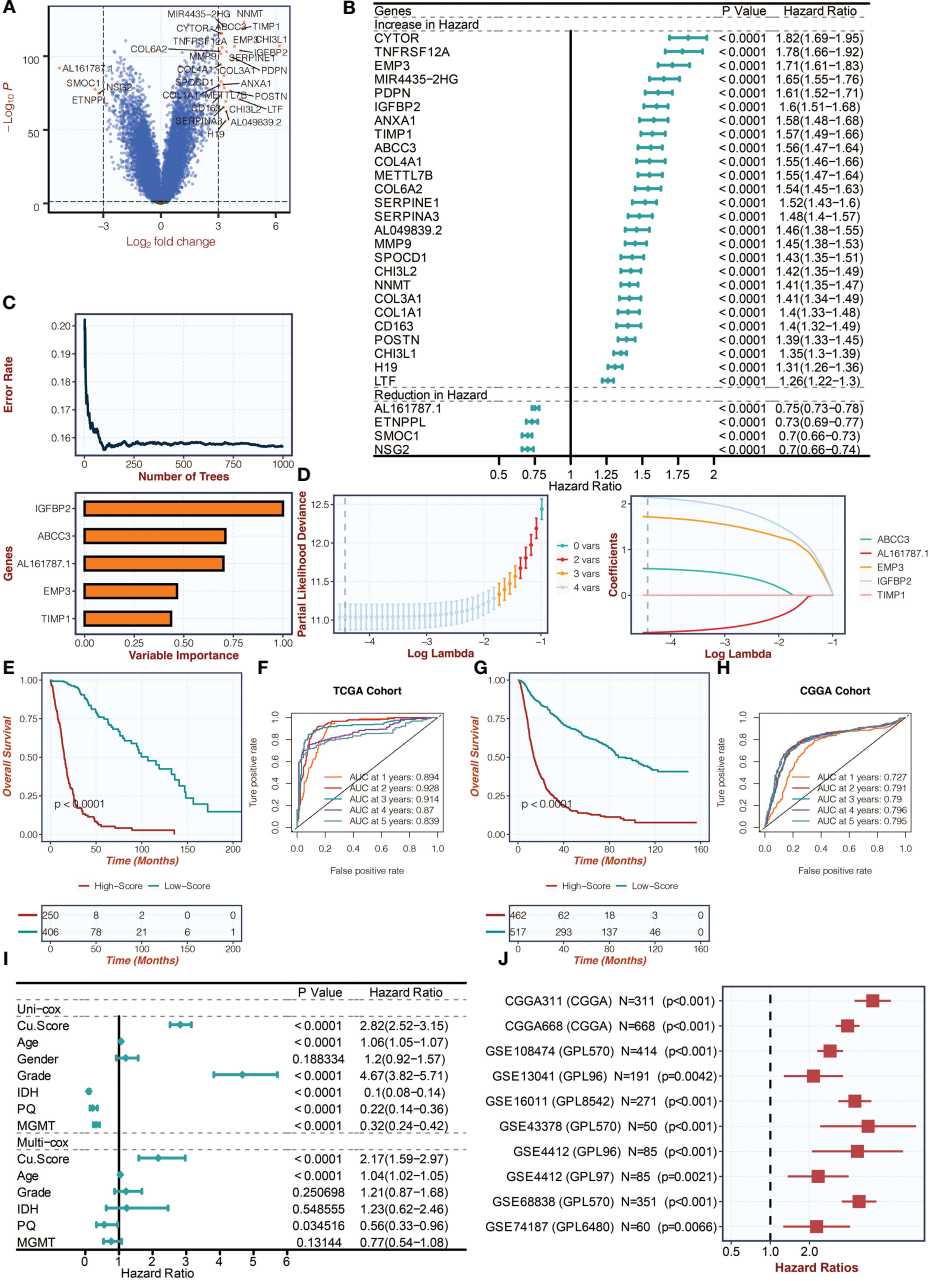
Creation of the copper metabolism-associated biomarker. **(A)** A volcano plot was created to show the differentially expressed genes (DEGs) between the copper metabolism-related clusters. **(B)** Univariate Cox regression analysis was conducted on the feature genes in the TCGA cohort. **(C)** The Random Survival Forest algorithm was employed to reduce the dimensionality of the DEGs in the TCGA cohort. **(D)** The LASSO algorithm was used for dimension reduction of the feature genes and to construct the copper metabolism-associated biomarker in the TCGA cohort. **(E)** Survival curves were presented for the copper metabolism-associated biomarker groups in the TCGA cohort. **(F)** ROC curves were used to showcase the performance of the copper metabolism-associated biomarker in the TCGA cohort. **(G)** Survival curves were depicted for the copper metabolism-associated biomarker groups in the CGGA cohort. **(H)** ROC curves were used to illustrate the performance of the copper metabolism-associated biomarker in the CGGA cohort. **(I)** Univariate and multivariate Cox regression analysis was conducted on the clinical characteristics in the TCGA cohort. **(J)** Univariate Cox regression analysis was performed on the copper metabolism-associated biomarker in ten glioma cohorts.

**Figure 2A** in the study displays the differentially expressed genes (DEGs) between copper metabolism-related clusters. These feature genes then underwent univariate Cox regression analysis in **Figure 2B**. To reduce dimensionality, the Random Survival Forest algorithm was applied in **Figure 2C**, and the weights of the genes were determined. The LASSO algorithm was further used to construct the copper metabolism-associated biomarker in **Figure 2D**. The formula for the biomarker is provided, including the genes’ weights. Survival curves were generated for the biomarker groups in both the TCGA and CGGA cohorts in **Figures 2E, G**, respectively. ROC curves were also plotted to assess the performance of the biomarker in predicting one-year, two-year, three-year, four-year, and five-year survival in **Figures 2F, H**. The ROC curves for the TCGA and CGGA cohorts are provided with their corresponding values. The association of the biomarker with tumor grade, methylation status, and IDH wildtype gliomas is shown in **Figures S1A, S1B**. Univariate and multivariate Cox regression analyses were conducted to confirm the biomarker’s independent prognostic value in **Figure 2I**. Additionally, the biomarker’s prognostic significance was evaluated across ten glioma cohorts in **Figure 2J**.

### The copper metabolism-associated biomarker could predict mutation patterns

**Figure 3:**
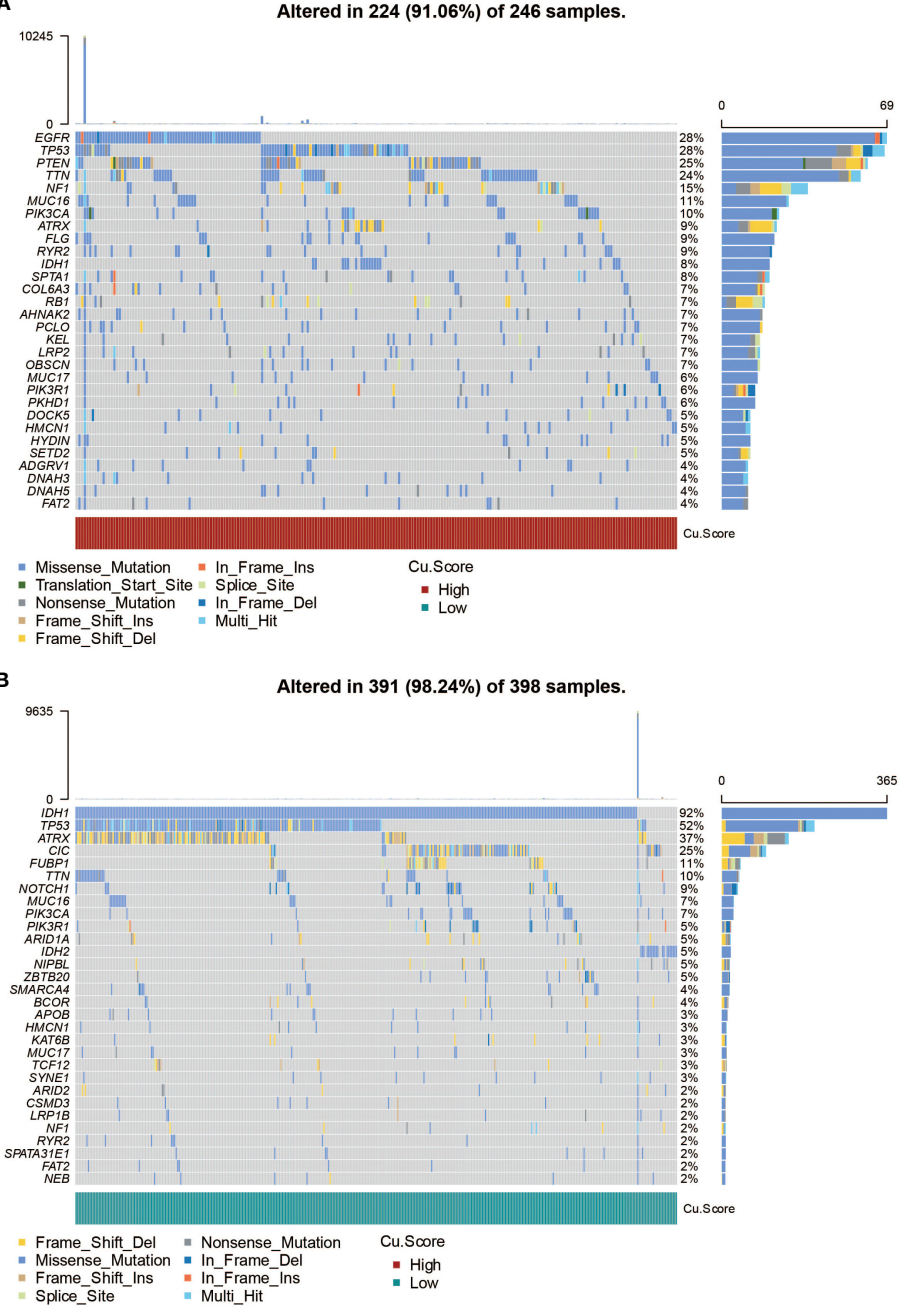
SNP analysis of the copper metabolism-associated biomarker. **(A)** A waterfall plot was generated to visualize the mutation frequency of genes in the high copper metabolism-associated biomarker group in the TCGA cohort. **(B)** Similarly, a waterfall plot was created to illustrate the mutation frequency of genes in the low copper metabolism-associated biomarker group in the TCGA cohort.

**Figure 4:**
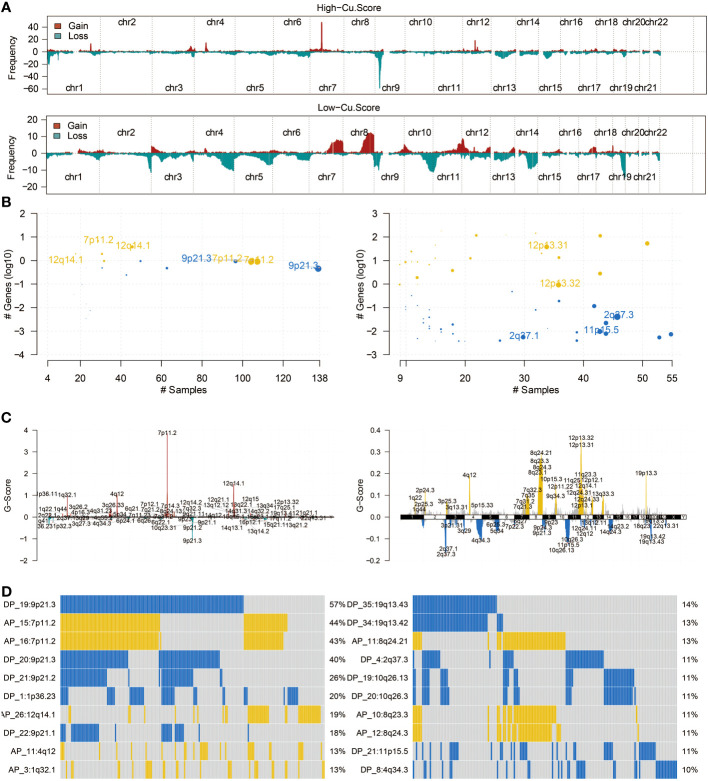
CNV analysis of the copper metabolism-associated biomarker. **(A)** An overview of the altered chromosomes in the two copper metabolism-associated biomarker groups in the TCGA cohort was provided. **(B)** A bubbleplot was used to represent the altered chromosomes in the two copper metabolism-associated biomarker groups in the TCGA cohort. **(C)** A chromplot was used to represent the altered chromosomes in the two copper metabolism-associated biomarker groups in the TCGA cohort. **(D)** An oncoplot was used to demonstrate the altered chromosomes in the two copper metabolism-associated biomarker groups in the TCGA cohort.

**Figure 3A** in the study presents the mutation frequency of genes in the high copper metabolism-associated biomarker group. This group’s top five mutated genes are EGFR, TP53, PTEN, TTN, and NF1. **Figure 3B** shows the mutation frequency in the low copper metabolism-associated biomarker group, with IDH1, TP53, ATRX, CIC, and FUBP1 as the most frequently mutated genes. Additionally, differentially mutated genes and their co-occurrences were observed in the two biomarker groups, such as IDH1, EGFR, PTEN, CIC, ATRX, TP53, NF1, NOTCH1, FUBP1, and RB1 (**Figures S2A**, **B**). Furthermore, **Figure 4A** illustrates the altered chromosomes in the two biomarker groups. **Figures 4B-D** present specific altered chromosomes and their frequencies within the high and low copper metabolism-associated biomarker groups. In the high copper metabolism-associated score group, chromosome 7p11.2 was amplified, and chromosome 9p21.3 was deleted. Notably, 9p21.3 had the highest frequency of changes (57%) in the genome in the group with high copper metabolism-related scores. The EGFR gene, located on chromosome 7p11.2, is involved in cell growth, proliferation, and survival, and mutations or overexpression of EGFR have been associated with tumor growth and progression. CDKN2A, located on chromosome 9p21.3, regulates cell division and prevents tumor formation. The consistent findings provide support for the reliability of the copper metabolism-associated biomarker.

### The copper metabolism-associated biomarker could predict immunotherapy response

**Figure 5:**
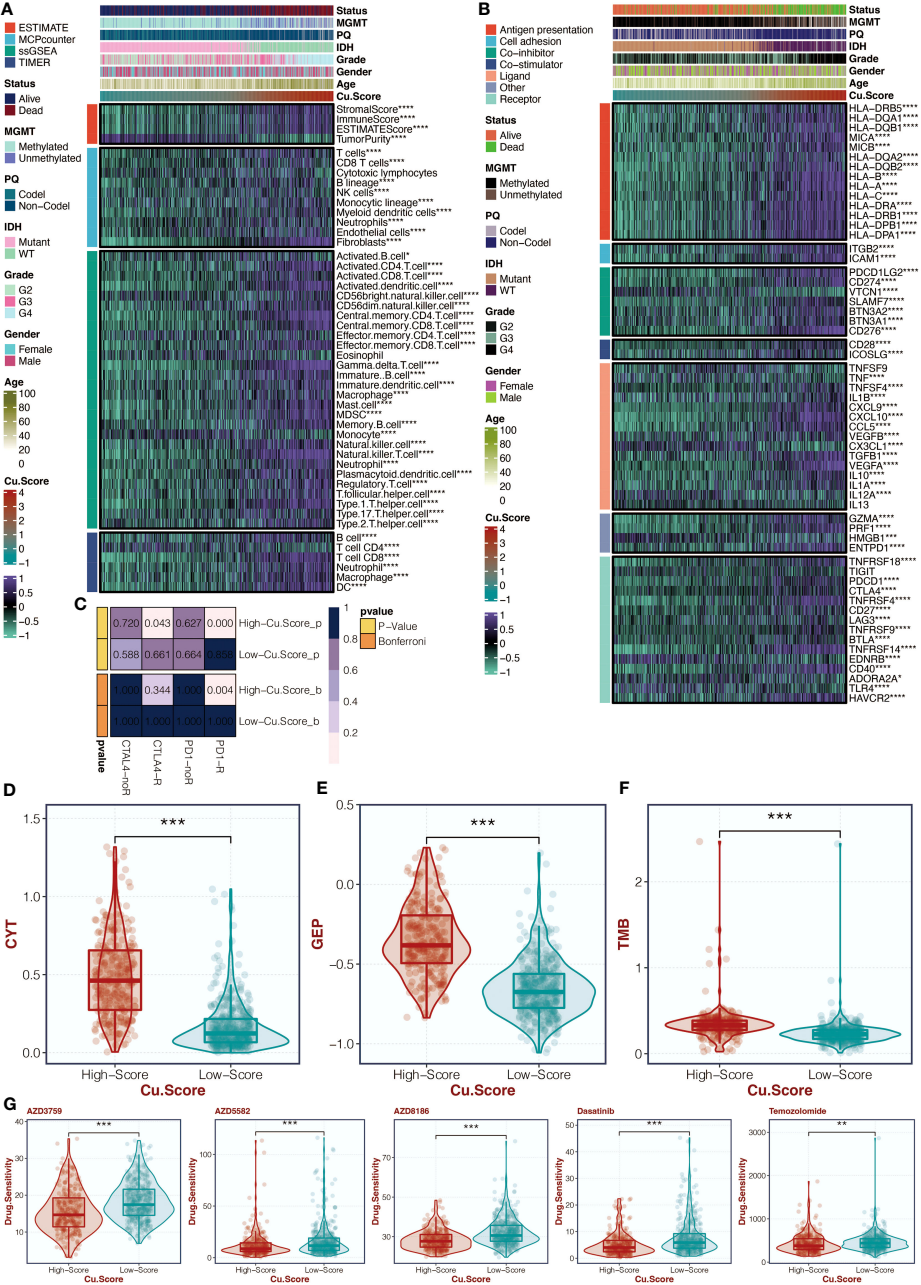
Immune analysis of the copper metabolism-associated biomarker. **(A)** A heatmap can be generated to display the correlation between the copper metabolism-associated biomarker and immune infiltrating cells in the TCGA cohort. **(B)** Another heatmap can be created to show the correlation between the copper metabolism-associated biomarker and immune modulators in the TCGA cohort. **(C)** TIDE analysis can be performed to reveal immunotherapy responses in the two copper metabolism-associated biomarker groups in the TCGA cohort. **(D)** A box plot can be used to showcase the levels of CYT in the two copper metabolism-associated biomarker groups in the TCGA cohort. **(E)** Another box plot can be created to depict the levels of GEP in the two copper metabolism-associated biomarker groups in the TCGA cohort. **(F)** A box plot can be generated to display the levels of TMB in the two copper metabolism-associated biomarker groups in the TCGA cohort. **(G)** Another box plot can be used to illustrate the levels of chemotherapy agents in the two copper metabolism-associated biomarker groups in the TCGA cohort. *, P<0.05; **, P<0.01; ***, P<0.001; ****, P<0.0001.

**Figures 5A, B** in the study examined the correlation between the copper metabolism-associated biomarker, immune infiltrating cells, and immune modulators. The results showed that CD4 T cells, CD8 T cells, B cells, neutrophils, macrophages, and dendritic cells were more active in the high copper metabolism-associated score group. Additionally, TNFRSF18, PDCD1, CTLA4, TNFRSF4, CD27, LAG3, TNFRSF14, BTLA, CD40, and HAVCR2 were more expressed in the high copper metabolism-associated score group. The TIDE analysis suggested that the high copper metabolism-associated biomarker group was more likely to respond to specific immunotherapies (**Figure 5C**). Furthermore, this group exhibited higher CYT, GEP, and TMB levels (**Figures 5D-F**). **Figure 5G** displayed the levels of chemotherapeutic drugs in the two groups based on copper metabolism-related scores in the TCGA cohort. The high copper metabolism-related score group showed decreased drug sensitivity to AZD3759, AZD5582, AZD8186, Dasatinib, and Temozolomide.

### The copper metabolism-associated biomarker could predict tumorigenic processes and immune activity

**Figure 6:**
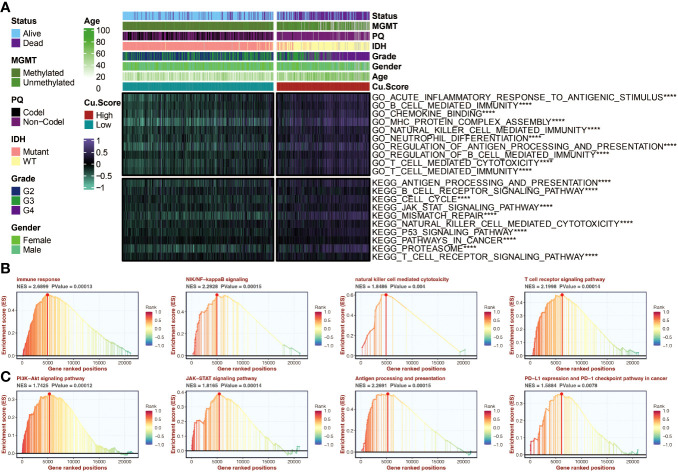
Functional annotation of the copper metabolism-associated biomarker. **(A)** A heatmap can be generated to display the correlation between the copper metabolism-associated biomarker and GSVA-based GO and KEGG pathways in the TCGA cohort. **(B)** GSEA of GO pathways related to the copper metabolism-associated biomarker. **(C)** GSEA of KEGG pathways linked to the copper metabolism-associated biomarker. ****, P<0.0001.

The copper metabolism-associated biomarker was positively associated with various immune, inflammation, and tumor-related pathways (**Figure 6A**). Enrichment analysis using GSEA revealed high enrichment in immune response and relevant pathways, such as the T cell receptor pathway and antigen presentation process (**Figures 6B, C**).

### IGFBP2, as a prognostic marker, could predict tumorigenic processes and immune activity

**Figure 7:**
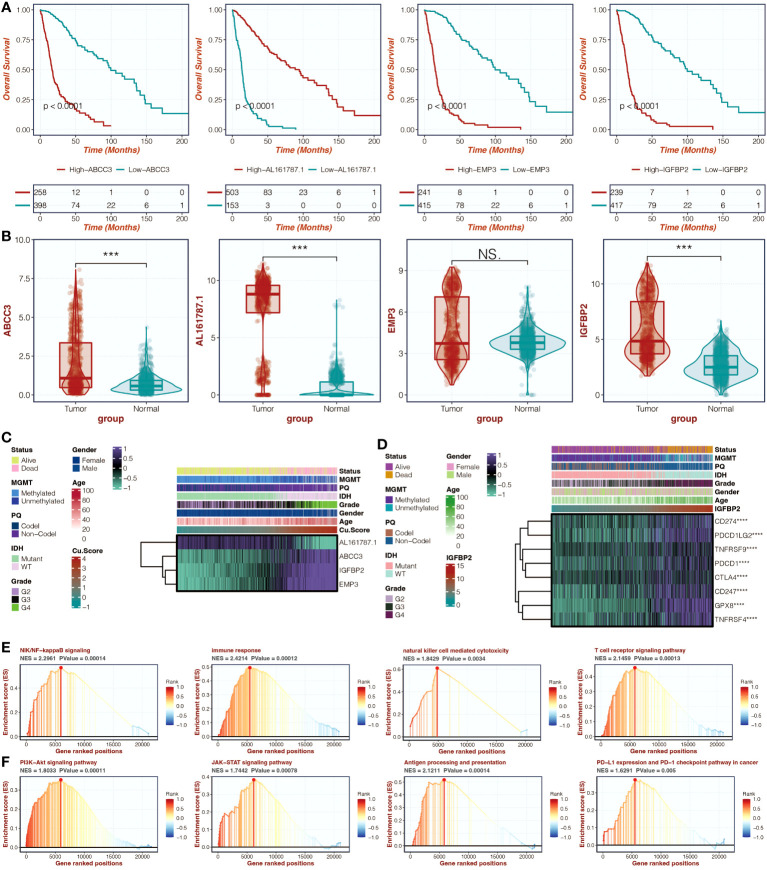
The molecular features of IGFBP2. **(A)** Survival curves of four model genes in the TCGA cohort can be generated to analyze their impact on patient survival. **(B)** A box plot can be created to compare the expression differences of four model genes between tumor samples and normal samples in the TCGA cohort. **(C)** A heatmap can be generated to visualize the expression differences of four model genes in the two copper metabolism-associated biomarker groups in the TCGA cohort. **(D)** A heatmap can be created to show the correlation between IGFBP2 and immune modulators in the TCGA cohort. **(E)** Gene Set Enrichment Analysis (GSEA) can be performed to identify Gene Ontology (GO) pathways related to IGFBP2. **(F)** GSEA can also be conducted to identify KEGG pathways related to IGFBP2. ***, P<0.001; ****, P<0.0001.

Survival curves for four model genes (ABCC3, EMP3, AL161787.1, and IGFBP2) highlighted IGFBP2 as a significant marker of decreased survival outcomes in glioma patients (**Figure 7A**). Its expression differences between tumor and normal samples were also evident (**Figure 7B**). **Figure 7C** illustrates the even distribution of expression differences of four model genes in the two groups based on copper metabolism-related scores in the TCGA cohort. In the TCGA cohort, **Figure 7D** shows the relationship between IGFBP2 and immune modulators. IGFBP2 had a significant positive correlation with all immune modulators. GSEA analysis of GO pathways on IGFBP2 revealed strong enrichment of pathways related to immune response, natural killer cell mediated cytotoxicity, and T cell receptor signaling (**Figure 7E**). The GSEA analysis of KEGG pathways showed high enrichment of the JAK-STAT signaling pathway and PI3K-Akt signaling pathway on IGFBP2 (**Figure 7F**).

### IGFBP2 could promote tumor progression and mediate macrophage migration

**Figure 8:**
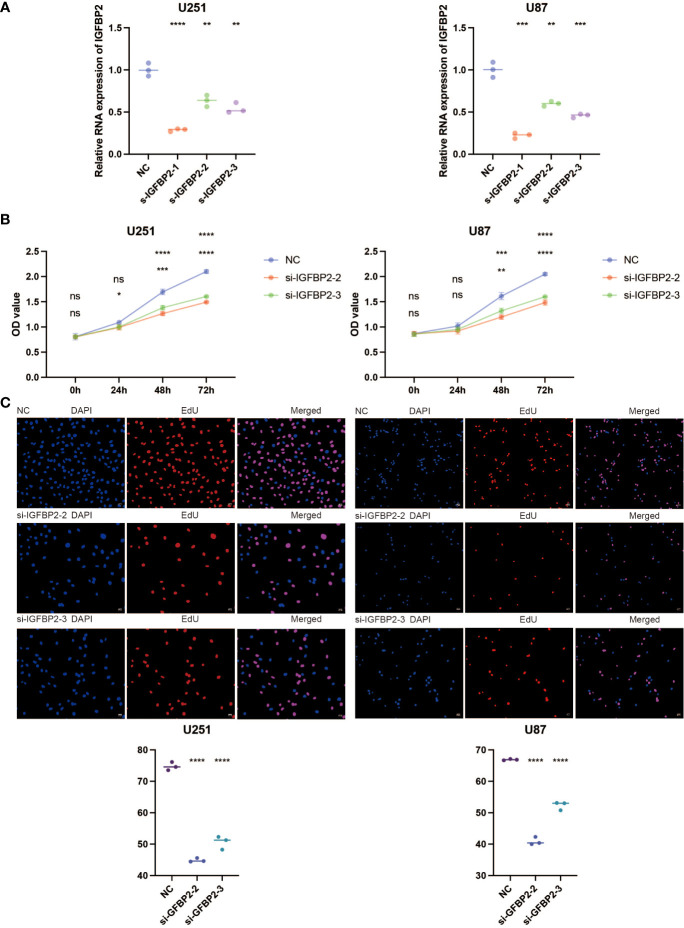
The tumor-promoting ability of IGFBP2. **(A)** qPCR assay conducted in four groups (NC, si-IGFBP2-1, si-IGFBP2-2, si-IGFBP2-3) in U251 and U87. **(B)** CCK-8 assay performed in three groups (NC, si-IGFBP2-2, si-IGFBP2-3) in U251 and U87. **(C)** EdU assay carried out in three groups (NC, si-IGFBP2-2, si-IGFBP2-3) in U251 and U87. *, P<0.05; **, P<0.01; ***, P<0.001; ****, P<0.0001. ns, not statistically significant.

**Figure 9:**
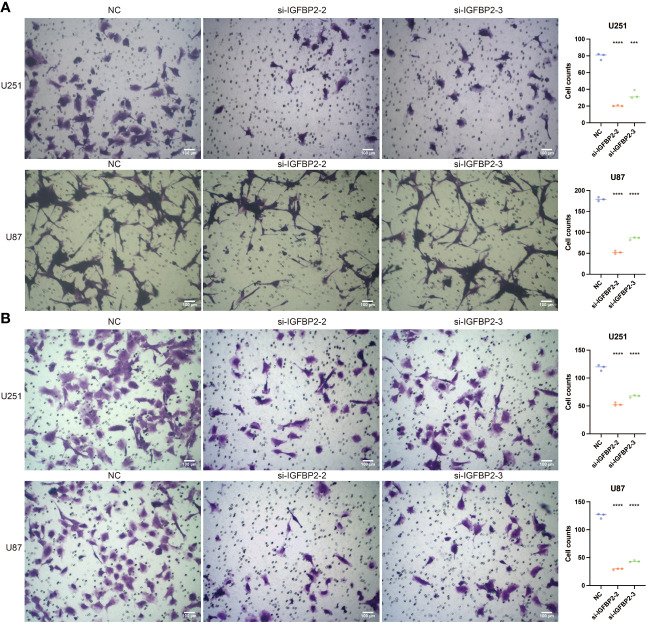
The tumor-promoting ability and macrophage-promoting ability of IGFBP2. **(A)** Transwell assay performed in three groups (NC, si-IGFBP2-2, si-IGFBP2-3) in U251 and U87. **(B)** Transwell assay conducted in three groups (NC, si-IGFBP2-2, si-IGFBP2-3) in HMC3 cocultured with U251 and U87. ***, P<0.001; ****, P<0.0001.

Further research was done on the possible tumor- and macrophage-promoting properties of IGFBP2. Three siRNA were tested using a q-PCR assay to determine their capacity to reduce IGFBP2 expression (**Figure 8A**). Two si-IGFBPs are more effective at inhibiting IGFBP2 expression: si-IGFBP2-2 and si-IGFBP2-3. In the si-IGFBP2-2 and si-IGFBP2-3 groups, the OD values of U251 and U87 were dramatically decreased (**Figure 8B**). In the si-IGFBP2-2 and si-IGFBP2-3 groups, there was a considerable decrease in the positively stained cells of U251 and U87 (**Figure 8C**). In the si-IGFBP2-2 and si-IGFBP2-3 groups, the number of U251 and U87 migrated cells was dramatically decreased (**Figure 9A**). The positively stained HMC3 cells in the si-IGFBP2-2 and si-IGFBP2-3 groups dramatically decreased after coculturing with U251 and U87 (**Figure 9B**). These findings demonstrated IGFBP2’s potential to actively encourage GBM migration and proliferation. Additionally, IGFBP2 may actively encourage macrophage migration.

### IGFBP2 is a critical microenvironment mediator by sc-RNA seq analysis

**Figure 10:**
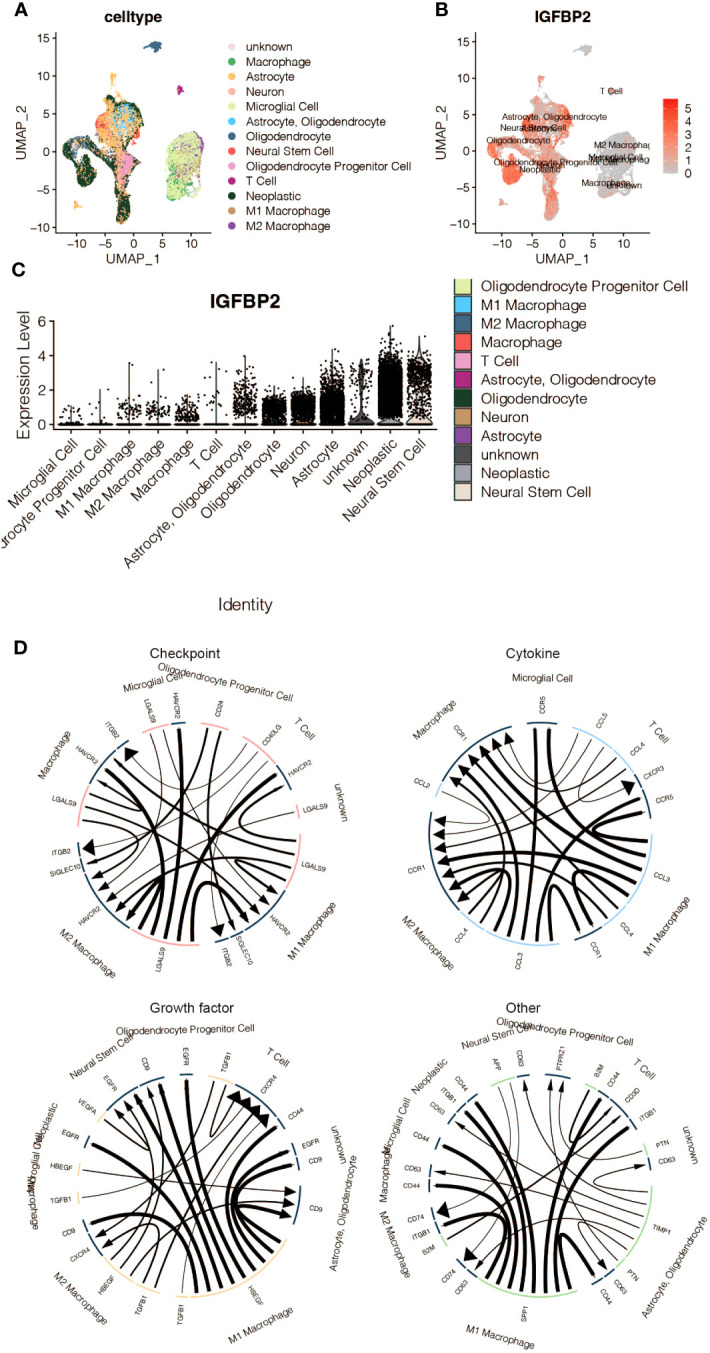
The immune characteristics of IGFBP2 at the scRNA-seq level. **(A)** UMAP representing different microenvironment cells. **(B)** UMAP representing the expression of IGFBP2 in different microenvironment cells. **(C)** Vlnplot representing the expression of IGFBP2 in different microenvironment cells. **(D)** The cell communication pattern concerning different signaling pathways in microenvironment cells expressing high IGFBP2.

**Figures 10A-D** in the study used sc-RNA seq analysis to explore the regulatory role of IGFBP2 in macrophages. The analysis identified expression clusters and cell communication in microenvironment cells that expressed high levels of IGFBP2. Specifically, UMAP and Vlnplot showed that IGFBP2 was highly expressed in neoplastic cells, neural stem cells, and astrocytes. The cell communication pattern suggested potential mechanisms of IGFBP2 in interacting with macrophages through specific signaling pathways, including EGFR, CD44, CD63, and ITGB1.

## Discussion

The body carefully maintains a delicate balance of copper content, as a deficiency and an excess can have significant consequences. Copper is essential for the proper functioning of metal-binding enzymes, and any abnormal levels can disrupt cellular processes and potentially result in cell death(22). Maintaining this delicate balance is crucial; numerous factors regulate copper intake, discharge, and metabolism to ensure homeostasis. When this balance is disrupted, it can result in abnormal copper metabolism and cell death, leading to the development of various diseases (2, 22). Interestingly, studies have revealed a correlation between copper metabolism and tumorigenesis, specifically in cancers that exhibit a higher demand for copper compared to healthy cells (4). For instance, breast cancer (23), cervical cancer (24), and glioma (25) have all exhibited altered copper content and distribution throughout the body. Therefore, it becomes crucial to understand the molecular features of copper metabolism-related genes in gliomas to unravel their significance.

Copper metabolism plays a crucial role in various cellular processes, including nucleotide metabolism. Copper ions serve as cofactors for specific enzymes involved in nucleotide synthesis, enabling important reactions such as the conversion of ribonucleotides (RNA building blocks) into deoxyribonucleotides (DNA building blocks) mediated by ribonucleotide reductase. These deoxyribonucleotides are vital for DNA synthesis and repair, which are fundamental to cell proliferation and immune response.

In cancer and immunotherapy, the connection between copper metabolism and nucleotide metabolism becomes particularly significant. Tumor cells often change their metabolic requirements to support rapid growth and proliferation, which increases the demand for nucleotides. Copper-dependent enzymes, such as copper-zinc superoxide dismutase (CuZnSOD), produce superoxide radicals that serve as crucial electron donors to ribonucleotide reductase during the conversion of ribonucleotides to deoxyribonucleotides. As a result, the availability of copper can affect the activity of ribonucleotide reductase, affecting the production of deoxyribonucleotides necessary for DNA synthesis in tumor cells.

The study aimed to create clusters related to copper metabolism based on genes associated with copper metabolism. These clusters significantly impacted the survival outcomes of glioma patients, effectively dividing them into distinct prognostic groups. Using machine learning algorithms such as Random Survival Forest and LASSO, a strong four-gene biomarker associated with copper metabolism was developed to predict prognosis. This biomarker was independent of age and 1p/19q status and outperformed them in predicting hazard. Furthermore, certain oncogenes like EGFR, PTEN, and TTN had a high frequency of mutations in the group with a high copper metabolism-associated biomarker, suggesting their potential relevance for therapeutic intervention in GBM (26) (27). On the other hand, tumor suppressors like IDH, TP53, and ATRX displayed a high mutation frequency in the low copper metabolism-associated biomarker group, and their significance in predicting tumor progression and behavior has been previously reported (28, 29).

The tumor microenvironment (TME) in gliomas is known to play a crucial role in tumor progression and recurrence, as mentioned in a study by Ma et al. in 2018 (30). This study observed that the high copper metabolism-associated biomarker group had higher microenvironment scores, including ESTIMATE, Immune, and Stromal scores. Despite gliomas traditionally being considered “cold” tumors with a relatively suppressed immune response due to the unique characteristics of the central nervous system, recent evidence suggests that immune system elements still operate in the tumor microenvironment (TME) of gliomas due to the disruption of the blood-brain barrier. Interestingly, the copper metabolism-associated biomarker was positively associated with several immune infiltrating cells, including macrophages, mast cells, MDSCs (myeloid-derived suppressor cells), neutrophils, regulatory T cells, and fibroblasts. Furthermore, immune response-related pathways, such as natural killer cell-mediated cytotoxicity, T cell receptor signaling, and NIK/NF-kappaB signaling, were highly enriched in the high copper metabolism-associated biomarker group. These findings suggest that targeting the immune suppressive microenvironment could potentially enhance immunotherapy responses in gliomas, particularly in the context of immune checkpoint inhibitors, which have shown promise in promoting anti-glioma immune responses. This information is based on a study by Ghouzlani et al. in 2021 (31).

Further examination of chemotherapy agents revealed that the high copper metabolism-associated biomarker group exhibited lower sensitivity to several drugs, including AZD3759, AZD5582, AZD8186, Dasatinib, and Temozolomide. These drugs have demonstrated the potential to inhibit glioma growth, but their effectiveness may be compromised by a high copper metabolism-associated biomarker. For example, AZD3759 has been reported to inhibit glioma by blocking the EGFR and JAK pathways (32). Enhanced activity of AZD5582 in rabies virus glycoprotein-lactoferrin-liposomes could downregulate inhibitors of apoptosis proteins in GBM (33). Inhibiting PI3Kβ with AZD8186 was revealed to regulate critical metabolic pathways in PTEN-null tumors (34). Dasatinib has indeed been extensively studied in preclinical research for its potential use in glioma treatment. Recent studies have shown that Dasatinib-induced autophagy is enhanced when combined with temozolomide in glioma. This suggests that the combination of Dasatinib and temozolomide may have synergistic effects in treating glioma (35). Temozolomide has always been the first-line treatment option for gliomas (36). IGFBP2, which promotes vasculogenic mimicry formation in glioma, could be considered a key player in tumorigenesis (37). It is overexpressed in several types of cancer, including breast, prostate, and colorectal cancer. IGFBP2 promotes tumor growth and metastasis by enhancing cell proliferation, survival, and migration (38). It can also contribute to angiogenesis, which is the formation of new blood vessels that supply nutrients to tumors (39). Additionally, IGFBP2 has been associated with resistance to chemotherapy and radiation therapy in certain cancers (40). Correspondingly, our study revealed that IGFBP2 could promote the proliferation and migration of gliomas.

IGFBP2 has been shown to regulate the activity of immune cells, such as macrophages and T cells, in the tumor microenvironment (41). Our study revealed that glioma-derived IGFBP2 could facilitate the migration of macrophages. The cell communication pattern suggested potential mechanisms of IGFBP2 interacting with macrophages through EGFR, CD44, CD63, and ITGB1 signaling pathways. These pathways have been previously shown to contribute to the polarization of macrophages towards a pro-tumoral phenotype, promoting tumor invasion and metastasis.

Additionally, IGFBP2 can modulate the expression of certain immune-related genes and cytokines, which are important for immune response and inflammation. It has also been suggested that IGFBP2 may play a role in immune evasion by tumors (42), as it can suppress the activity of natural killer cells, which are important for immune surveillance against cancer cells. Interestingly, highly expressing IGFBP2 samples showed enrichment in immune response-related pathways, indicating a potential immunogenic role for IGFBP2 in glioma. Additionally, IGFBP2 was found to be a potent immunotherapy determinant. It was discovered that IGFBP2 controls PD-L1 expression in malignant melanoma by activating the EGFR-STAT3 signaling pathway (43).

In conclusion, the study highlights the importance of copper metabolism in glioma patients. The study shows that copper metabolism is interconnected with nucleotide metabolism, as specific nucleotide synthesis enzymes rely on copper ions. Copper is essential for electron transfer and enzyme activity, which are necessary for accurate nucleotide synthesis. Nucleotides play a crucial role in various cellular processes. Although some studies have already tried to establish prognostic models in gliomas (44–46), our study has some advantages. We used machine learning algorithms to ensure the reliability of the model. Besides, we systematically explored the potential of this biomarker in prognosis, mutation analysis, and predicting immunotherapy response efficiency in gliomas. Furthermore, not only the oncogenic role but also the immunogenic role of IGFBP2 was explored by *in vitro* validation.

The copper metabolism-associated biomarker constructed in this research shows promise for clinical applications and understanding the regulatory mechanisms of copper metabolism in gliomas. It can also help differentiate glioma patients with different outcomes and potentially guide targeted immunotherapy and chemotherapy treatments. The clinical implications of IGFBP2 are significant as well. Targeting IGFBP2 could be a potential therapeutic strategy for treating gliomas by inhibiting its activity and slowing down tumor growth. IGFBP2 could also serve as a biomarker for diagnosing and monitoring the progression of gliomas. Additionally, IGFBP2 may enhance the tumor-promoting effects of immune cells, leading to increased inflammation, angiogenesis, and immunosuppression within the tumor microenvironment. Targeting IGFBP2 with combined immunotherapy could have therapeutic value.

However, there are some limitations to the study. Validation in a real-world cohort is needed to confirm the copper metabolism-related clusters and biomarker. More machine learning algorithms could increase the reliability and application of the biomarker. Future *in vitro* and *in vivo* experiments are also necessary to validate the specific tumorigenic and immunogenic mechanisms of IGFBP2 in gliomas and advance glioma research.

## Data availability statement

The original contributions presented in the study are included in the article/**Supplementary Material**. Further inquiries can be directed to the corresponding author.

## Ethics statement

Ethical approval was not required for the studies on humans in accordance with the local legislation and institutional requirements because only commercially available established cell lines were used.

## Author contributions

QL: Data curation, Investigation, Methodology, Software,Writing – original draft. JZ: Investigation, Software, Writing – original draft. DZ: Data curation, Investigation, Writing – original draft. CM: Supervision, Writing – review & editing. HL: Data curation, Software, Writing – original draft. JP: Data curation, Writing – original draft. CZ: Methodology, Writing – original draft. CQ: Software, Writing – original draft. CL: Investigation, Writing – original draft. MC: Data curation, Writing – original draft. YX: Supervision, Writing – review & editing. DH: Supervision, Writing – review & editing. ZC: Funding acquisition, Supervision, Writing – original draft, Writing – review & editing.
