# Supplementary material for: IGFBP2 from a novel copper metabolism-associated biomarker promoted glioma progression and response to immunotherapy
Source: Front Immunol. 2023 Oct 19;14:1282734. doi: 10.3389/fimmu.2023.1282734 (PMC10620745; doi:10.3389/fimmu.2023.1282734)
Supplement: Supplementary file 1 [file DataSheet_1.docx]

Supplementary methods

Cell culture

The human U251, U87, and HMC3 cell lines were purchased from iCell (http://www.icellbioscience.com/search). U251 and U87 cells were cultured in DMEM medium containing 10% fetal bovine serum (FBS) and 1% double antibody at 37℃ with 5% CO2 in an incubator with saturated humidity. HMC3 cells were cultured in 1640 medium containing 10% FBS and 1% double antibody under the same conditions.

q-PCR assay

The protocol for the qPCR assay involves several steps, including sample preparation, reverse transcription, PCR amplification, and data analysis. Primers were designed using Primer 5.0. The primer sequences for β-actin and IGFBP2 were as follows: β-actin (F: ACCCTGAAGTACCCCATCGAG; R: AGCACAGCCTGGATAGCAAC) with a product length of 224bp and IGFBP2 (F: TTGTGAGAAGCGCCGGGAC; R: GCCTCCTTCTGAGTGGTCATC) with a product length of 82bp.

Cell Counting Kit-8 (CCK8) assay

The cells were digested and counted in 96-well plates at a density of 2*103 cells/well, 100 μL per well. After the culture adhered to the wall, the corresponding treatment time was carried out according to the above method. Then, 10μl/well of CCK8 (Abiowell) was added to each well, and the CCK8 solution was configured with a complete medium. The absorbance (OD) at 450nm was analyzed by Bio-Tek enzyme label after incubating at 37℃ with 5% CO2 for 2 h.

EdU assay

An appropriate EdU (Ribo, C10310) medium of 50μM was prepared by diluting the EdU solution (reagent A) with cell culture medium at 1000:1. Cells were cultivated overnight in each well after 100μL of 50 m EDU media had been added. PBS was applied to the cells 1–2 times for 5 minutes. Each well received 50 mL of cell fixative (4% paraformaldehyde), which was then incubated for 30 minutes at room temperature. Each well received 50 mL of 2 mg/mL glycine, which was then incubated for 5 minutes in a shaker. Each well was treated with 100μL of PBS and washed in the decolorization shaker for 5 minutes. 100μL of penetrant was added to each well for decolorization and incubated in the shaker for 10 minutes. 100 μL of 1x Apollo® staining solution was added to each well and incubated in a shaker at room temperature for 30 minutes, away from light. The shaker was cleaned 2-3 times with 100 μL of penetrant, each time for 10 minutes. Each well was cleaned 1-2 times with 100 μL of methanol for 5 minutes. Reagent F was diluted with deionized water at a ratio of 100:1 to prepare 1x Hoechst33342 reaction solution. 100 μL of 1x Hoechst 33342 reaction solution was added to each well and incubated in a shaker for 30 minutes at room temperature, away from light. Each well was treated with 100 μL of PBS 1-3 times. The observation was conducted immediately after staining.

Transwell assay

Matrigel was diluted with 100ul of cold, serum-free DMEM medium per well for a final concentration of 200ug Matrigel. In the lower compartment, 500ul 10% FBS complete medium was added. The cells were digested by trypsin into single cells, then re-suspended to 2*106 cells /ml in a serum-free medium, with 100ul cells added to each well (Corning). For 48 hours, the cells were incubated at 37°C. After being cleaned with PBS three times, the upper chamber was fixed for 20 minutes with 4% paraformaldehyde. The upper chamber was stained for 5 minutes with 0.1% crystal violet and then five times with water. An inverse microscope was used to examine the surface cells.

The U251 and U87 cells were placed in the lower chamber with 500ul 10% FBS complete medium, and the HMC3 cells were placed in the upper chamber with serum-free DMEM medium. The follow-up procedures were the same as mentioned above.

Supplementary Figures


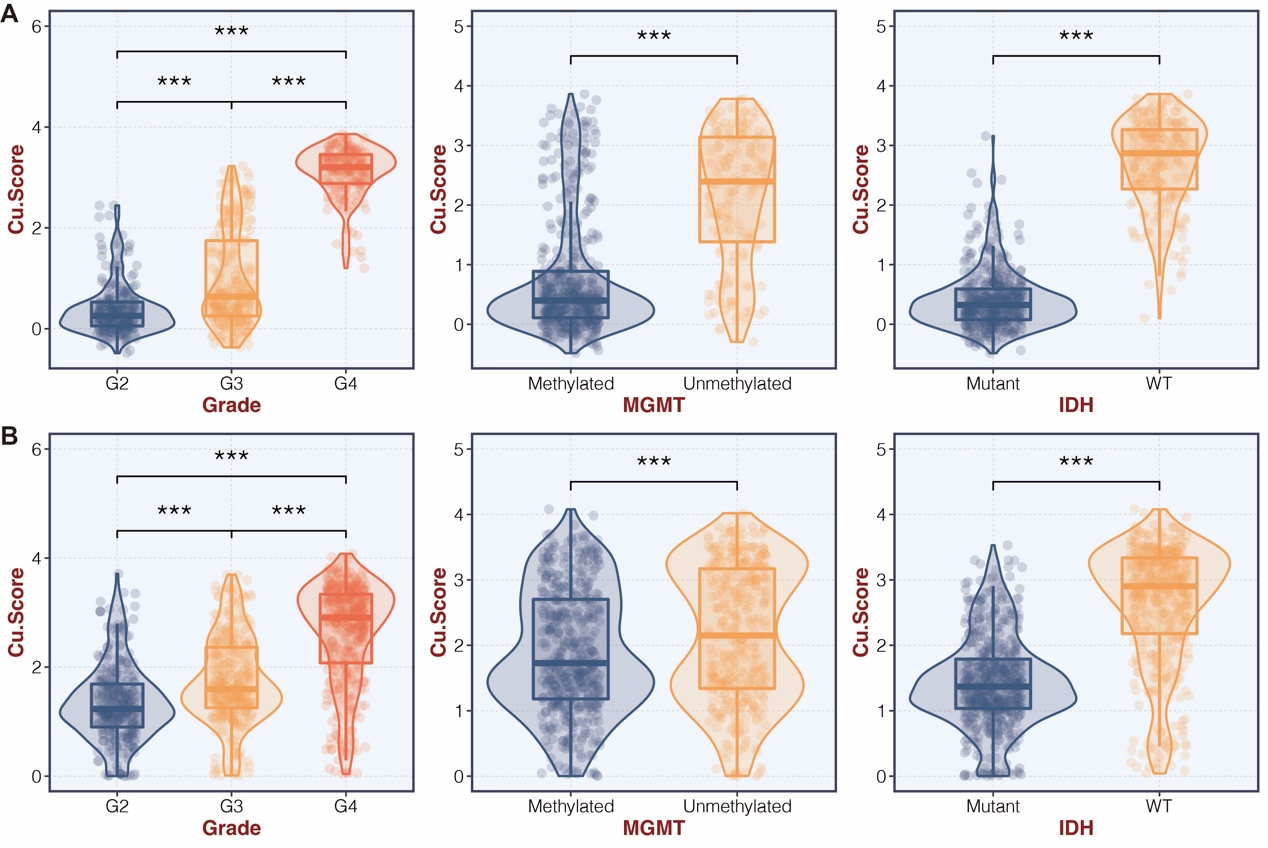


Figure S1. The molecular features of copper metabolism-related score. A. Box plot showing the expression differences of tumor grade, MGMT status, and IDH status in the two copper metabolism-related score groups in the TCGA cohort. B. Box plot showing the expression differences of tumor grade, MGMT status, and IDH status in the two copper metabolism-related score groups in the CGGA cohort.


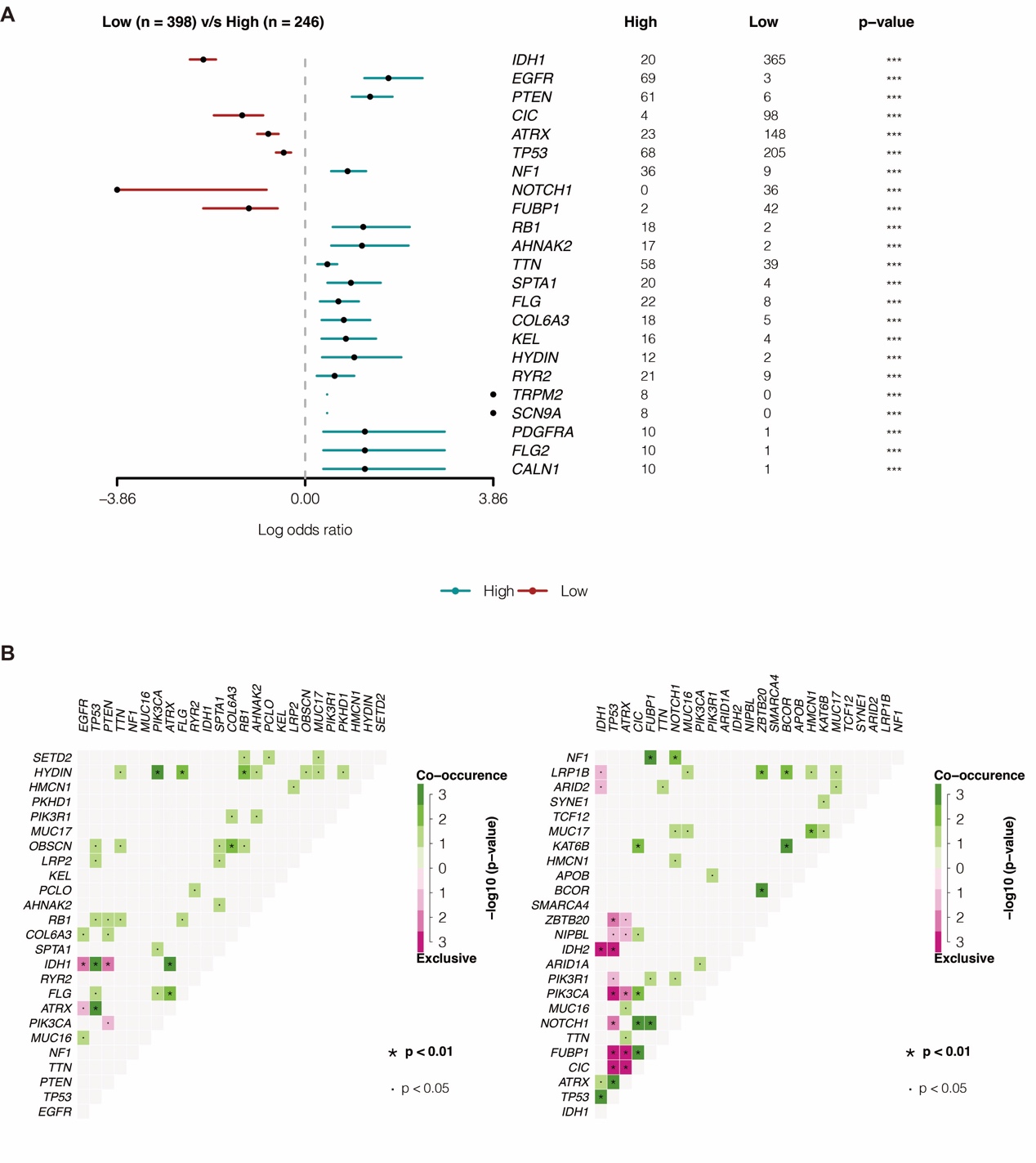


Figure S2. SNP analysis of the copper metabolism-related score. A. The differentially mutated genes in the two copper metabolism-related score groups. B. The mutually occurred and exclusive mutations in the two copper metabolism-related score groups.
